# Optical Coherence Tomography Angiography-Derived Peripapillary Vessel Density Findings in Multiple Sclerosis

**DOI:** 10.3390/jcm15041329

**Published:** 2026-02-07

**Authors:** Angeliki G. Filippatou, Vasilios S. Liarakos, Eirini Okoutsidou, Dimitrios Tzanetakos, Aikaterini Theodorou, Lina Palaiodimou, Maria-Ioanna Stefanou, Alexandra Akrivaki, Evangelia-Makrina Dimitriadou, John S. Tzartos, Sotirios Giannopoulos, Konstantinos Voumvourakis, Georgios Tsivgoulis

**Affiliations:** 1 Second Department of Neurology, “Attikon” University Hospital, School of Medicine, National and Kapodistrian University of Athens, 124 62 Athens, Greece; 2 Department of Ophthalmology, Naval Hospital, 115 21 Athens, Greece; 3 First Department of Ophthalmology, “G. Gennimatas” General Hospital, School of Medicine, National and Kapodistrian University of Athens, 115 27 Athens, Greece

**Keywords:** multiple sclerosis, optical coherence tomography, retina, optic nerve, optic neuritis

## Abstract

**Background/Objectives:** Multiple sclerosis (MS)-related optic neuritis (ON) results in thinning of the peripapillary nerve fiber layer (pRNFL) which tends to be temporal quadrant-predominant. Optical coherence tomography angiography (OCTA) enables visualization of the retinal vasculature. Prior studies have shown reduced peripapillary vessel density (VD) in MS but data on the quadrantic pattern of peripapillary VD loss are limited. Our objective was to investigate the pattern of OCTA-derived peripapillary VD reduction in MS. **Methods:** People with MS (PwMS) and healthy controls (HC) underwent optic disc OCTA scans (Solix, Optovue) and VD was derived for the peripapillary region and quadrants. Eyes with ON within six months were excluded. Analyses were performed with generalized estimating equations models and standardized coefficients are presented. **Results:** We included 50 eyes from 29 PwMS (12 ON, 38 non-ON) and 12 eyes from 6 HC. VD in the peripapillary region was lower in MS ON eyes compared to HC with the largest effect size observed in the temporal quadrant (average: −1.47, *p* < 0.001; superior: −1.08, *p* = 0.006; inferior: −0.94; *p* = 0.017; temporal: −1.55; *p* < 0.001; nasal: −1.06, *p* = 0.007). In MS non-ON eyes, only temporal VD was significantly lower compared to HC eyes (temporal: −0.77, *p* = 0.004). Moderate to strong correlations were observed between OCT and corresponding OCTA metrics from the same regions. **Conclusions:** Our findings suggest that vascular alterations in the peripapillary region may exhibit a temporal quadrant predominant pattern. Larger studies are needed to further characterize the patterns and temporal evolution of retinal peripapillary vascular injury in MS.

## 1. Introduction

Multiple sclerosis (MS) is a demyelinating disease of the central nervous system [1]. Involvement of the anterior visual pathway is common, and approximately 50% of people with MS (PwMS) experience optic neuritis (ON) during the course of their disease. In ON, there is inflammatory demyelination of the optic nerve, which results in injury and transection of the axons of the retinal ganglion cells (RGCs). Consequently, there is retrograde degeneration of the RGC axons and cell bodies which results in thinning of the retinal nerve fiber layer (RNFL; containing the RGC axons that coalesce at the optic disc to form the optic nerve) and of the ganglion cell layer (containing the RGC cell bodies). Even in the absence of a clinical history of ON, eyes of PwMS commonly demonstrate subclinical optic neuropathy, a finding that can be detected through imaging and electrophysiological testing and has been corroborated by histopathological evidence [2,3].

Structural changes in the retina can be quantified in vivo via the use of retinal optical coherence tomography (OCT). OCT is a non-invasive, rapid and reproducible imaging modality that provides high-resolution images of the retina by use of near-infrared light. Extensive literature has shown that MS optic neuropathy results in thinning of the peripapillary RNFL (pRNFL) and of the macular ganglion cell layer, typically measured as composite with the inner plexiform layer (GCIPL) due to technical reasons [2]. Prior meta-analytic evidence indicates that, relative to healthy controls (HCs), pRNFL thickness is, on average, 18–20 μm lower in eyes of PwMS with a history of ON, and GCIPL thickness is, on average, approximately 16 μm lower [2,4]. In MS eyes without a clinical history of ON, pRNFL and GCIPL thickness are each, on average, approximately 7 μm lower relative to HCs [2]. Overall, OCT enables the detection and quantification of retinal neuro-axonal damage in MS.

The optic nerve was recently added to the MS McDonald diagnostic criteria as a fifth topographical location to demonstrate dissemination in space. Optic nerve involvement can be established based on different structural and functional modalities, including orbital magnetic resonance imaging (MRI), retinal OCT or visual evoked potentials [5,6]. OCT studies have specifically focused on inter-eye differences in retinal layer thicknesses given the propensity for asymmetric thinning of the pRNFL and the GCIPL in PwMS relative to HCs. Asymmetry is common when only one eye has been affected by ON but has also been observed in PwMS with no known history of ON in either eye; these inter-eye differences are likely due to subclinical optic neuropathy and correlate with inter-eye differences in low-contrast visual acuity scores, indicating potential functional implications [7]. According to the 2024 McDonald MS diagnostic criteria, OCT-derived inter-eye differences of at least 6 μm for pRNFL or 4 μm for GCIPL support unilateral optic nerve involvement and can be used to objectively demonstrate optic nerve involvement for the diagnosis of MS, provided no better explanation exists and rigorous OCT quality control has been applied [6]. These thresholds were based on multi-center validation studies and have been reproduced across different OCT platforms.

Interestingly, pRNFL thinning secondary to MS-related optic neuropathy tends to be temporal quadrant predominant, which is the region of the pRNFL containing the papillomacular bundle [4,6]. The papillomacular bundle contains RNFL axons projecting from the macular RGCs to the optic nerve and is rich in parvocellular axons that are hypothesized to be more sensitive to demyelination attacks [8]. This may be due to unique metabolic demands of these axons but the precise underlying pathophysiological mechanisms are incompletely understood.

OCT angiography (OCTA) is a newer non-invasive technology derived from conventional OCT that enables visualization of the retinal vasculature. OCTA detects erythrocyte motion by composing sequential OCT B-scans [9]. This is achieved by leveraging the variation in OCT signal caused by moving particles (namely erythrocytes) as the contrast mechanism for imaging blood flow in perfused retinal blood vessels [10]. OCTA can be used to visualize the retinal vascular plexuses, including the peripapillary vascular network. OCTA has been incorporated into ophthalmology clinical practice, and a growing body of literature has reported alterations in retinal microvasculature in neuroinflammatory conditions [9,11,12]. In MS, prior studies have suggested that peripapillary vessel density (VD) and macular VD are reduced in eyes with a history of ON and, to a lesser extent, in eyes without a clinical history of ON [4,9]. However, studies evaluating the quadrantal pattern of peripapillary VD loss—particularly examining whether it follows a temporal-predominant pattern similar to pRNFL thinning—are limited.

In this study, we aimed to investigate the pattern of OCTA-derived peripapillary VD reduction in MS eyes with and without a history of ON. We hypothesized that peripapillary VD would be reduced in both MS ON and MS non-ON eyes compared to HCs, and that these reductions would be temporal-predominant.

## 2. Methods

### 2.1. Study Participants

We retrospectively screened people with relapsing–remitting MS who had undergone OCTA for inclusion in the present study. Eligibility required a confirmed MS diagnosis in accordance with the 2017 revised McDonald diagnostic criteria [13]. History of ON was defined clinically at the discretion of the treating clinician, based on whether the patient had experienced symptoms consistent with ON in that eye [14]. Eyes that had experienced ON within six months were excluded because post-ON changes remain dynamic and structural loss is still evolving during this interval, whereas this process largely stabilizes by six months [15]. HCs were included among individuals who underwent routine ophthalmologic assessments and had no identifiable ocular pathology. Exclusion criteria for both PwMS and HCs included glaucoma, refractive errors greater than 6 diopters, or other ophthalmologic or neurologic disorders that may interfere with OCT testing or results.

### 2.2. OCT/OCTA Methods

OCT scans were acquired using the Optovue SOLIX device (Fremont, CA, USA). Scans were acquired without pupillary dilation using the Disc Cube protocol for optic disc scans and the Retina Cube protocol for macular scans. The Disc Cube protocol includes 350 B-scans equally spaced covering 6 mm *y*-axis, each with 350 A-scans covering 6 mm *x*-axis, while the Retina Cube protocol includes 200 B-scans, each with 512 A-scans, covering a 6.4 × 6.4 mm region centered at the fovea. Scans with a scan quality indicator below 6 were excluded and quality control of OCT scans was performed according to the OSCAR-IB criteria [16]. Retinal segmentation was performed according to the integrated software for pRNFL and quadrants from the optic disc scans and for ganglion cell layer (GCC) from the macular scans. GCC is a composite layer comprising the macular RNFL and the GCIPL.

**Figure 1:**
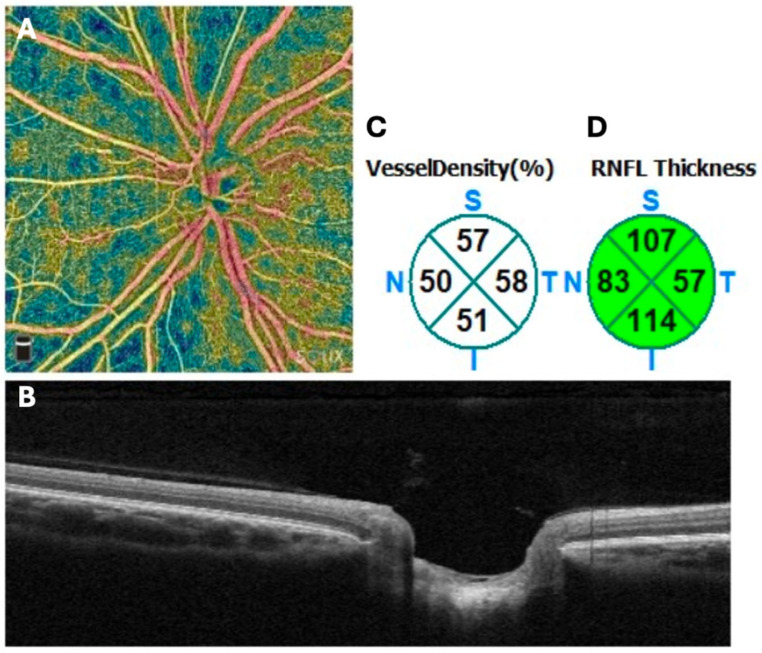
Example of peripapillary OCT and OCTA scan. Panel (**A**): En-face visualization of peripapillary vascular network with color-coded vessel density; Panel (**B**): Peripapillary OCT B-scan example; Panel (**C**): OCTA-derived peripapillary vessel density for superior (S), inferior (I), temporal (T) and nasal (N) quadrants (in %); Panel (**D**): OCT-derived peripapillary RNFL thickness for superior (S), inferior (I), temporal (T) and nasal (N) quadrants (in μm). *OCT: optical coherence tomography; OCTA: OCT angiography; RNFL: retinal nerve fiber layer*.

OCTA scans were acquired using the Optovue SOLIX device (Fremont, CA, USA). Scans were acquired without pupillary dilation using the AngioVue Disc protocol for optic disc. The protocol includes 512 B-scans, each with 6512 A-scans, covering a 6 × 6 mm region. Scans with a scan quality indicator below 6 were excluded and quality control of OCTA scans was performed according to the OSCAR-MP criteria [17]. Vessel density was derived according to the integrated software for the slab between the internal limiting membrane and outer boundary of the RNFL for the following regions: whole image, peripapillary region quadrants (nasal, temporal, inferior, superior) (Figure 1).

### 2.3. Statistical Methods

OCT and OCTA metrics were summarized using mean and standard deviation (SD). Comparisons between MS ON, MS non-ON and HC eyes were performed using generalized estimating equations with robust standard errors, accounting for within-subject, inter-eye correlations. Models with both non-standardized and standardized coefficients are presented. Standardized models were performed by converting each OCT metric to a z-score prior to analysis by subtracting the mean and dividing by SD. This transformation normalizes each metric to a mean of 0 and a standard deviation of 1, enabling direct comparison of effect sizes across metrics with different anatomical baseline values. Correlations between OCT and OCTA metrics were evaluated with Pearson’s correlation coefficients. Statistical analyses were performed using Stata version 18. Statistical significance was defined as *p* < 0.05 (two-tailed). Analyses were based on a priori established research hypotheses; consequently, adjustment for multiple comparisons was not performed; results should be interpreted cautiously as exploratory findings.

## 3. Results

In this study, we screened 33 people with relapsing–remitting MS for inclusion. One participant was excluded due to an unknown ON history and three were excluded due to OCTA scans failing quality control in both eyes, resulting in 29 included participants (16/29 [55%] female, mean age 43.6 ± 11.6). Among them, 12 PwMS had a prior history of unilateral ON, one had a prior history of ON in both eyes and 16 PwMS did not have a prior history of ON in either eye. After further exclusion of scans from eight eyes that failed quality control, a total of 50 eyes from PwMS were analyzed (12 ON and 38 non-ON eyes). We additionally included 12 eyes from 6 HCs (6/6 [100%] female, mean age 31.3 ± 3.5).

For MS participants, mean disease duration was 9.7 ± 7.3 years. Reported MS disease-modifying treatments at the time of the OCTA scan included teriflunomide (*n* = 3), cladribine (*n* = 6), ocrelizumab (*n* = 3), dimethyl fumarate (*n* = 3), fingolimod (*n* = 2), interferon beta-1a (*n* = 3), ozanimod (*n* = 3), natalizumab (*n* = 1), ofatumumab (*n* = 3), glatiramer acetate (*n* = 1), and none (*n* = 1). None of the participants had a history of diabetes mellitus; four had a history of controlled hypertension.

**Figure 2:**
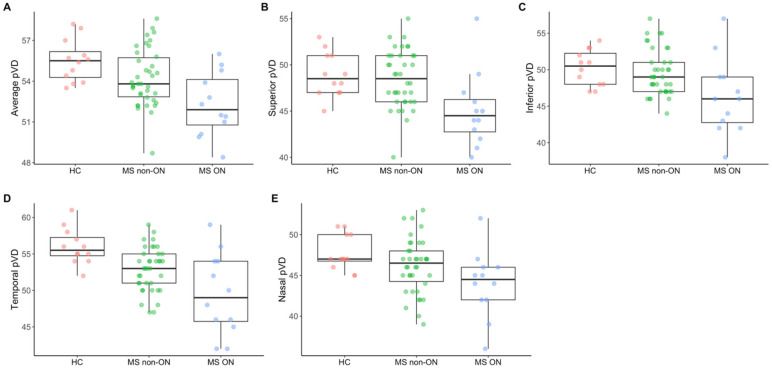
Peripapillary OCTA metrics in HC, MS non-ON and MS ON eyes. Panel (**A**): Average peripapillary vessel density; Panels (**B**–**E**): Peripapillary vessel density quadrants; (**B**): Superior; (**C**): Inferior; (**D**): Temporal; (**E**): Nasal. *OCTA: OCT angiography; pVD: peripapillary vessel density; HC: healthy controls; MS: multiple sclerosis; ON: optic neuritis*.

OCT and OCTA metrics in MS ON, MS non-ON eyes and HCs are summarized in Table 1. OCTA metrics are graphically illustrated in Figure 2. Comparisons between MS ON, MS non-ON and HC eyes derived from models with both non-standardized and standardized coefficients are presented in Table 2, Table 3 and Table 4. Only standardized coefficients are presented in the results section.

With regard to OCT metrics, average and quadrantal pRNFL thicknesses were lower in MS ON eyes compared to HC eyes with the largest effect size observed in the temporal quadrant and the lowest in the nasal quadrant (average: −1.83 [95% CI: −2.54 to −1.11], *p* < 0.001; superior: −1.63 [95% CI: −2.30 to −0.96], *p* < 0.001; inferior: −1.62 [95% CI: −2.37 to −0.88], *p* < 0.001; temporal: −1.89 [95% CI: −2.68 to −1.10], *p* < 0.001; nasal: −0.92 [95% CI: −1.75 to −0.09], *p* = 0.029) (Table 2). Similarly, average and quadrantal pRNFL thicknesses were lower in MS ON eyes compared to MS non-ON (average: −1.02 [95% CI: −1.56 to −0.47], *p* < 0.001; superior: −0.95 [95% CI: −1.44 to −0.47], *p* < 0.001; inferior: −0.99 [95% CI: −1.53 to −0.45], *p* < 0.001; temporal: −0.70 [95% CI: −1.22 to −0.17], *p* = 0.009; nasal: −0.65 [95% CI: −1.17 to −0.13], *p* = 0.014) (Table 3). Average, superior, inferior and temporal but not nasal pRNFL thicknesses were significantly lower in MS non-ON eyes compared to HC eyes, again with the largest effect size observed in the temporal quadrant (average: −0.81 [95% CI: −1.45 to −0.18], *p* = 0.012; superior: −0.67 [95% CI: −1.33 to −0.02], *p* = 0.044; inferior: −0.64 [95% CI: −1.22 to −0.06], *p* = 0.032; temporal: −1.19 [95% CI: −1.84 to −0.54], *p* < 0.001; nasal: −0.28 [−1.09 to 0.54], *p* = 0.51) (Table 4).

With regard to OCTA metrics, average peripapillary and quadrantal VD was lower in MS ON eyes compared to HC eyes with the largest effect size observed in the temporal quadrant (average: −1.47 [95% CI: −2.17 to −0.77], *p* < 0.001; superior: −1.08 [95% CI: −1.84 to −0.32], *p* = 0.006; inferior: −0.94 [95% CI: −1.70 to −0.17], *p* = 0.017; temporal: −1.55 [95% CI: −2.40 to −0.70], *p* < 0.001; nasal: −1.06 [95% CI: −1.83 to −0.29], *p* = 0.007) (Table 2). Average peripapillary and quadrantal VD was lower in MS ON eyes compared to MS non-ON with generally comparable effect sizes between quadrants (average: −0.92 [95% CI: −1.36 to −0.47], *p* < 0.001; superior: −0.93 [95% CI: −1.58 to −0.27], *p* = 0.006; inferior: −0.76 [95% CI: −1.40 to −0.12], *p* = 0.019; temporal: −0.78 [95% CI: −1.48 to −0.08], *p* = 0.029; nasal: −0.62 [95% CI: −1.23 to −0.004], *p* = 0.048) (Table 3). In MS non-ON eyes, only temporal VD was significantly lower compared to HC eyes (average: −0.56 [95% CI: −1.14 to 0.03], *p* = 0.06; superior: −0.15 [95% CI: −0.68 to 0.37], *p* = 0.57; inferior: −0.17 [95% CI: −0.59 to 0.24], *p* = 0.41; temporal: −0.77 [95% CI: −1.29 to −0.25], *p* = 0.004; nasal: −0.45 [95% CI: −1.04 to 0.15], *p* = 0.14) (Table 4).

Moderate to strong correlations were observed between OCT and corresponding OCTA metrics from the same regions (Table 5), with the strongest correlation observed in the temporal region (average: r = 0.71, *p* < 0.001; superior: r = 0.52, *p* < 0.001; inferior: r = 0.56, *p* < 0.001; temporal pRNFL vs. temporal VD: r = 0.76, *p* < 0.001; nasal: r = 0.50, *p* < 0.001).

## 4. Discussion

In this study, we investigated the pattern of OCTA-derived peripapillary VD reduction in MS. While our results should be interpreted with caution given the modest sample size, we found that peripapillary VD was globally reduced in MS ON eyes compared with both HC and MS non-ON eyes. Reductions were observed across all peripapillary quadrants, although the largest effect size was noted in the temporal quadrant. This vascular pattern closely paralleled our structural OCT findings. Previous studies using OCT have demonstrated a temporal predominance of structural damage in MS-related optic neuropathy, which has been attributed to the preferential involvement of the papillomacular bundle. The papillomacular bundle is rich in parvocellular axons, which are hypothesized to be more sensitive to demyelination attacks [6,8]. Although the precise mechanism has not been fully elucidated, the smaller size of parvocellular axons may contribute to differential susceptibility to inflammatory injury, possibly due to unique metabolic needs [8]. Our results appear to extend these prior observations, by suggesting that a similar temporal predominance may also characterize peripapillary vascular involvement in MS ON.

Subclinical optic nerve involvement is well recognized in MS. Thinning of the inner retinal layers is frequently detected by OCT even in eyes without a clinical history of ON, albeit these changes may be less pronounced than in eyes with a prior history of clinically evident symptomatic ON [2,6]. A substantial body of literature has utilized OCT to demonstrate reductions in pRNFL and macular GCIPL thickness in MS eyes without a history of ON; our findings are consistent with this [2,4]. With regard to OCTA findings, we observed that, in MS non-ON eyes, only peripapillary VD in the temporal VD quadrant was reduced compared to HCs. These results raise the possibility that the hypothesized temporal predilection for peripapillary microvascular change may extend to MS non-ON eyes. Additionally, MS ON eyes exhibited greater VD loss than non-ON eyes, consistent with a gradient of both structural and microvascular changes, with more pronounced alterations in clinically affected ON eyes. While these observations align with our study hypotheses, they should be interpreted with caution given the small sample size and retrospective study design; confirmation in larger, prospective cohorts is warranted.

While several studies have examined OCTA findings in eyes from PwMS, studies investigating quadrantic patterns of peripapillary VD reduction are limited [4,9,12]. Lee at al. found that VD in all peripapillary quadrants except the nasal were lower in MS ON eyes compared to HCs [18]. Ulusoy et al. reported lower peripapillary VD in the inferior and temporal quadrant in MS ON eyes compared to MS non-ON and HC eyes, whereas peripapillary VD did not differ between MS non-ON and HC eyes in any quadrant [19]. Yilmaz et al. reported that only the temporal quadrant peripapillary VD significantly differed between MS and HC eyes; however, the study did not distinguish eyes with and without prior ON [20]. In another study by Rogaczewska et al., VD in all peripapillary quadrants was lower in MS ON eyes compared to HCs, and in all quadrants except the nasal in MS non-ON compared to HCs [21]. In contrast to these findings, Cordon et al. found no significant differences in VD in any of the peripapillary quadrants between MS ON, MS non-ON and HC eyes [22]. Overall, most evidence points towards a temporal predominance of peripapillary VD loss in eyes from PwMS but findings are inconsistent between studies, highlighting the need for larger studies to draw definitive conclusions about the pattern of peripapillary VD loss in MS.

The mechanisms by which retinal vasculature is affected in MS have not been fully elucidated. One hypothesis is that reductions in peripapillary VD represent a secondary phenomenon due to retinal neuro-axonal loss and reduced metabolic demand in the setting of pRNFL atrophy. This is supported by a prior study utilizing macular OCTA that suggested that reductions in macular OCTA metrics may be a delayed phenomenon as compared to GCIPL atrophy [23]. In the present study, we observed moderate to strong correlations between structural OCT and OCTA metrics in corresponding retinal regions, which supports this hypothesis. Longitudinal studies are needed to further investigate the temporal relationship between retinal neuro-axonal loss and microvascular changes. Another possible hypothesis is primary vascular injury due to vasculopathy associated with MS. Possible mechanisms may involve retinal perivascular inflammation and endothelial dysfunction [3]. Some studies have suggested a partial dissociation between neurodegeneration and vascular perfusion in MS; for example, one study reported reduced grey matter perfusion in MS that appeared to occur independently of gray matter atrophy [24]. Lastly, the impact of vascular risk factors and systemic metabolic disease on retinal perfusion in MS is insufficiently characterized. These comorbidities may independently influence OCTA-derived metrics. While this was beyond the scope of the present study, future work is needed to investigate the effects of these factors.

This study has several limitations that warrant discussion. Firstly, our study was limited by its small sample size and retrospective cross-sectional design. Larger studies are needed to confirm our observations and more definitively characterize the patterns of peripapillary VD loss in MS. Secondly, our control group was small and differed in demographics from the MS cohort: HCs were exclusively female and younger in age. As a result, the differential impact of demographic factors could not be assessed. Based on prior literature, OCTA metrics may decline with increased age [25,26], but it seems unlikely that age differences alone would explain the magnitude of differences between MS and HCs observed in this study. The influence of sex on OCTA metrics is uncertain, with mixed findings reported in the literature [26,27]. At present, no reference database is available for Solix OCTA data, limiting our ability to contextualize our findings against established normative values. Additionally, this study focused on the quadrantal pattern of peripapillary VD loss in MS and was not designed to evaluate structure–function relationships. Metrics of MS disease severity (e.g., disability measures such as the Expanded Disability Status Scale) were not systematically available for the present cohort but evaluating the relationship between peripapillary OCTA metrics and clinical outcomes would be of interest for future work. Lastly, a comprehensive assessment of cardiovascular health was not available for our participants (e.g., smoking status or lipid profile). Since systemic vascular risk factors may influence OCTA measurements in PwMS, future studies should incorporate standardized assessments of cardiovascular health to investigate their effects.

Overall, while OCTA is not yet integrated into routine clinical practice in MS, it is a valuable research tool. OCTA enables the quantification of retinal microvascular changes and may provide complementary insights into the spectrum of retinal pathology in MS, with potential biomarker value beyond conventional structural OCT. OCTA metrics have been associated with disability, visual function and brain volumetrics in MS, suggesting potential links between retinal vasculature and global central nervous system processes [28,29]. However, OCTA is susceptible to artifacts and, given its motion-contrast methodology, may not fully differentiate between low-flow states and vessel rarefaction [10,30]. Despite these limitations, OCTA is a promising modality for further research in MS.

## 5. Conclusions

In summary, we investigated the pattern of OCTA-derived peripapillary VD reduction in MS. Our findings may contribute to the understanding of the spectrum of retinal involvement in MS, suggesting that both structural and vascular alterations in the peripapillary region may exhibit a temporal quadrant predominant pattern. Larger, prospective, longitudinal studies are needed to further characterize the patterns and temporal evolution of retinal vascular injury in MS and its clinical relevance.

## Figures and Tables

**Table 1:** OCT and OCTA findings in MS ON and non-ON eyes.

	MS ON (*n* = 12)	MS non-ON (*n* = 38) ^	HC (*n* = 12)
**OCT**			
Average pRNFL (μm)	70.75 (8.90)	82.06 (9.69)	91.58 (7.89)
pRNFL quadrants (μm)			
- Superior	86.75 (8.62)	101.31 (12.83)	110.33 (10.87)
- Inferior	88.92 (20.75)	108.58 (15.45)	121.75 (13.07)
- Temporal	44.67 (11.53)	51.97 (8.23)	65.92 (9.26)
- Nasal	64.33 (8.85)	70.44 (14.07)	74.92 (12.39)
Macular GCC * (μm)	82.92 (7.08)	94.64 (8.36)	-
**OCTA**			
Peripapillary VD (%)	52.28 (2.34)	54.24 (2.09)	55.51 (1.56)
pVD quadrants (%)			
- Superior	45.08 (4.01)	48.50 (3.12)	48.92 (2.39)
- Inferior	46.33 (5.19)	49.63 (3.13)	50.25 (2.45)
- Temporal	49.50 (5.55)	52.89 (3.00)	56.00 (2.45)
- Nasal	44.00 (4.05)	46.16 (3.32)	47.75 (2.18)

^ OCT not available for 2 MS non-ON eyes; * Macular GCC not available for HC eyes. Data presented as mean (standard deviation).

**Table 2:** Comparison between MS ON and HC eyes.

	MS ON vs. HC Non-Standardized Coefficients (95% CI)	MS ON vs. HC Standardized Coefficients (95% CI)
OCT		
Average pRNFL	−20.56 μm (−28.61 to −12.50); *p* < 0.001	−1.83 (−2.54 to −1.11); *p* < 0.001
pRNFL quadrants		
- Superior	−22.58 μm (−31.92 to −13.24); *p* < 0.001	−1.63 (−2.30 to −0.96); *p* < 0.001
- Inferior	−31.09 μm (−45.32 to −16.85); *p* < 0.001	−1.62 (−2.37 to −0.88); *p* < 0.001
- Temporal	−21.49 μm (−30.47 to −12.52); *p* < 0.001	−1.89 (−2.68 to −1.10); *p* < 0.001
- Nasal	−12.13 μm (−23.03 to −1.22); *p* = 0.029	−0.92 (−1.75 to −0.09); *p* = 0.029
OCTA		
Peripapillary VD	−3.34% (−4.92 to −1.75); *p* < 0.001	−1.47 (−2.17 to −0.77); *p* < 0.001
pVD quadrants		
- Superior	−3.71% (−6.34 to −1.09); *p* = 0.006	−1.08 (−1.84 to −0.32); *p* = 0.006
- Inferior	−3.49% (−6.34 to −0.63); *p* = 0.017	−0.94 (−1.70 to −0.17); *p* = 0.017
- Temporal	−6.26% (−9.67 to −2.84); *p* < 0.001	−1.55 (−2.40 to −0.70); *p* < 0.001
- Nasal	−3.66% (−6.32 to −1.01); *p* = 0.007	−1.06 (−1.83 to −0.29); *p* = 0.007

**Table 3:** Comparison between MS ON and MS non-ON eyes.

	MS ON vs. MS Non-ON Non-Standardized Coefficients (95% CI)	MS ON vs. MS Non-ON Standardized Coefficients (95% CI)
OCT		
Average pRNFL	−11.43 μm (−17.59 to −5.27); *p* < 0.001	−1.02 (−1.56 to −0.47); *p* < 0.001
pRNFL quadrants		
- Superior	−13.23 μm (−20.01 to −6.45); *p* < 0.001	−0.95 (−1.44 to −0.47); *p* < 0.001
- Inferior	−18.90 μm (−29.20 to −8.61); *p* < 0.001	−0.99 (−1.53 to −0.45); *p* < 0.001
- Temporal	−7.95 μm (−13.93 to −1.97); *p* = 0.009	−0.70 (−1.22 to −0.17); *p* = 0.009
- Nasal	−8.51 μm (−15.30 to −1.73); *p* = 0.014	−0.65 (−1.17 to −0.13); *p* = 0.014
Macular GCC	−11.87 μm (−17.49 to −6.25); *p* < 0.001	−1.25 (−1.84 to −0.66); *p* < 0.001
OCTA		
Peripapillary VD	−2.08% (−3.09 to −1.07); *p* < 0.001	−0.92 (−1.36 to −0.47); *p* < 0.001
pVD quadrants		
- Superior	−3.18% (−5.44 to −0.92); *p* = 0.006	−0.93 (−1.58 to −0.27); *p* = 0.006
- Inferior	−2.84% (−5.23 to −0.46); *p* = 0.019	−0.76 (−1.40 to −0.12); *p* = 0.019
- Temporal	−3.16% (−5.99 to −0.32); *p* = 0.029	−0.78 (−1.48 to −0.08); *p* = 0.029
- Nasal	−2.12% (−4.23 to −0.02); *p* = 0.048	−0.62 (−1.23 to −0.004); *p* = 0.048

**Table 4:** Comparison between MS non-ON and HC eyes.

	MS Non-ON vs. HC Non-Standardized Coefficients (95% CI)	MS Non-ON vs. HC Standardized Coefficients (95% CI)
OCT		
Average pRNFL	−9.13 μm (−16.25 to −2.01); *p* = 0.012	−0.81 (−1.45 to −0.18); *p* = 0.012
pRNFL quadrants		
- Superior	−9.35 μm (−18.44 to −0.26); *p* = 0.044	−0.67 (−1.33 to −0.02); *p* = 0.044
- Inferior	−12.18 μm (−23.31 to −1.05); *p* = 0.032	−0.64 (−1.22 to −0.06); *p* = 0.032
- Temporal	−13.54 μm (−20.92 to −6.17); *p* < 0.001	−1.19 (−1.84 to −0.54); *p* < 0.001
- Nasal	−3.61 μm (−14.31 to 7.08); *p* = 0.51	−0.28 (−1.09 to 0.54); *p* = 0.51
OCTA		
Peripapillary VD	−1.26% (−2.59 to 0.06); *p* = 0.06	−0.56 (−1.14 to 0.03); *p* = 0.06
pVD quadrants		
- Superior	−0.53% (−2.35 to 1.29); *p* = 0.57	−0.15 (−0.68 to 0.37); *p* = 0.57
- Inferior	−0.65% (−2.19 to 0.90); *p* = 0.41	−0.17 (−0.59 to 0.24); *p* = 0.41
- Temporal	−3.10% (−5.20 to −1.00); *p* = 0.004	−0.77 (−1.29 to −0.25); *p* = 0.004
- Nasal	−1.54% (−3.58 to 0.50); *p* = 0.14	−0.45 (−1.04 to 0.15); *p* = 0.14

**Table 5:** Correlations between OCT and OCTA metrics.

Average pRNFL vs. Average VD	r = 0.71, *p* < 0.001
Superior pRNFL vs. Superior VD	r = 0.52, *p* < 0.001
Inferior pRNFL vs. Inferior VD	r = 0.56, *p* < 0.001
Temporal pRNFL vs. Temporal VD	r = 0.76, *p* < 0.001
Nasal pRNFL vs. Nasal VD	r = 0.50, *p* < 0.001

## Data Availability

Data from this study may be requested from the corresponding author, upon reasonable request. The data are not publicly available due to privacy/ethical restrictions.
